# Increasing Air Temperatures and Its Effects on Growth and Productivity of Tomato in South Florida

**DOI:** 10.3390/plants9091245

**Published:** 2020-09-21

**Authors:** Ibukun T. Ayankojo, Kelly T. Morgan

**Affiliations:** Institute of Food and Agricultural Sciences, South West Florida Research and Education Center, University of Florida, Immokalee, FL 34142, USA; conserv@ufl.edu

**Keywords:** climate change, heat stress, planting dates, temperature simulation, tomato yield

## Abstract

Florida ranks first among US states in fresh-market tomato production with annual production exceeding one-third of the total annual production in the country. Although tomato is a signature crop in Florida, current and future ambient temperatures could impose a major production challenge, especially during the fall growing season. This problem is increasingly becoming an important concern among tomato growers in south Florida, but studies addressing these concerns have not been conducted until now. Therefore, this study was conducted to determine the impacts of the present ambient temperature conditions and planting dates on tomato productivity in south Florida. The study was conducted using crop simulation model CROPGRO-Tomato of DSSAT (Decision Support System for Agricultural Transfer) version 4.7. Five treatments were evaluated, and included AT (simulated treatment using 14 years of actual daily weather conditions at the study location) while other treatments were conducted based on a percentage (−20%, −10%, +10%, +20%) of AT to simulate cooler and warmer temperature regimes. The results suggested that under the current temperature conditions during the fall growing season in south Florida, average tomato yield was up to 29% lower compared to the cooler temperature regimes. Tomato yield further decreased by 52% to 85% at air temperatures above the current condition. Yield reduction under high temperature was primarily due to lower fruit production. Contrary to yield, both tomato biomass accumulation and leaf area index increased with increase in temperature. Results also indicated that due to changes in air temperature pattern, tomato yield increased as planting date increased from July to December. Therefore, planting date modification during the fall season from the current July–September to dates between November and December will reduce the impacts of heat stress and increase tomato productivity in south Florida.

## 1. Introduction

Fresh-market tomato is a major vegetable crop in Florida with the total planted area of nearly 11,000 ha and total cash value of about $336 million in 2018 [1]. Florida ranks first among US states in fresh-market tomato production with annual production exceeding one-third of the total annual production in the country. Tomato production in Florida could be significantly affected by high temperature or heat stress, especially in the south Florida production area. This is because a major proportion of a typical tomato production season (under the current weather condition) in this region may be outside the optimum temperature regimes for maximum crop performance. This condition, however, may be worsened in the nearest future, especially as the projected global mean air temperature is expected to increase between 2.6 and 4.8 °C by the end of the twenty-first century [2].

According to Singh et al. [3], high temperature is considered as one of the most detrimental environmental factors affecting agriculture. Increasing temperature affects plant growth and reproduction, hence leading to significant losses in productivity. Plants physiological responses to heat stress have been extensively studied. Both transitory and constant exposure of plants to heat stress have effects on the physiology, morphology, and biochemical activities in plants that involves changes in cell structure and metabolism and alterations in the accumulation of protein as well as primary and secondary metabolites [4,5]. Heat stress is also known to cause oxidative stress in plants through the production of reactive oxygen species such as singlet oxygen (^1^O_2_), superoxide ion (O_2_), hydrogen peroxide (H_2_O_2_), and hydroxyl radicals (OH) with major damages to plant growth and general performances [6].

Generally, plant reproductive stages such as flowering and fruit setting are more sensitive to temperature stress than vegetative stages [7]. A typical problem associated with temperature extremes during the reproductive stages of tomato production is blossom drop, a problem often experienced by tomato growers in Florida [8]. Although tomato is grown under a wide range of climatic conditions, they are extremely sensitive to hot and humid environments [9], which is typical of most tomato production areas in south Florida. Under a high temperature condition, the fertility rate of tomato flowers is greatly reduced, leading to flower drop and reduced fruit setting. Tomato plants drop flowers when exposed to several days of daytime temperature above 29 °C and nighttime temperature above 21 °C, while reproduction is severely affected at day temperatures above 35 °C [10]. Flower drop can occur within just four hours of tomato exposure to daytime temperature above 40 °C [8].

Optimum range of air temperature for tomato production varies across several studies and tomato developmental stages. Depending on the developmental stage, the optimal mean daily range of air temperatures for tomato are between 21 to 24 °C [11], while 22 to 28 °C has been reported as tomato optimum low and high temperatures, respectively [12,13]. An increase of a few degrees above the optimal temperature range can result in a reduction in fruit set, and thus fruit production [14]. Therefore, any additional warming of the climate may be detrimental to tomato production [15], especially in south Florida with a pattern of increasing air temperatures over the last decade during the winter months (data not included).

**Figure 1:**
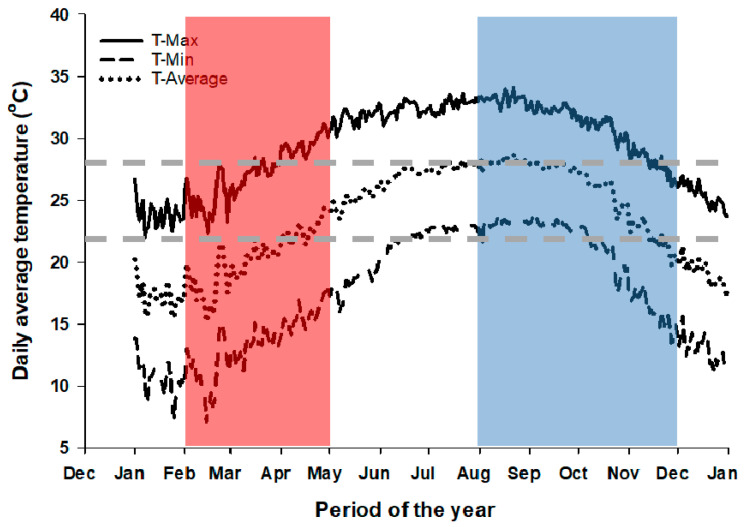
Average daily minimum, maximum, and mean temperatures from 2000 to 2017 in a typical South Florida tomato production area (Immokalee). Data generated from the Florida Automated Weather Network (FAWN). Shaded portions on the graph indicate typical spring (red) and fall (blue) tomato production seasons in south Florida from planting to harvesting. The horizontal broken lines indicate the optimum threshold for mean daily temperature for tomato crop.

In Florida, tomato production nearly covers the entire state extending from south to north; however, most production occurs towards south Florida [16]. In south Florida, tomato production typically occurs during two major growing seasons (spring and fall). The cooler spring season is typically from February to May, while the fall season is generally between August and December (Figure 1). For most growers in south Florida, the planting season during fall starts from mid to late August. However, planting season can be as early as late July to early August for some growers in this region [17].

The mean daily air temperature during a typical fall growing season in south Florida can be as high as 28 °C with the daily high temperature reaching 35 °C (Figure 1). These high temperatures could persist for most of the growing season, especially during the fall growing season (Figure 1). Therefore, high temperature could be a major abiotic stress for Florida tomato production for both current and future climatic conditions. Although the effects of high temperature on open-field fresh-market tomato during the fall growing season has become of increasing concern among many growers in south Florida, no study has addressed these concerns until now. Therefore, this study was conducted to: (1) determine the effect of increasing ambient temperature (simulated during flowering and fruit setting) on growth and yield of fresh market tomato crop under a typical Florida production condition, and (2) identify the optimum planting dates for tomato production during the fall growing season in south Florida.

## 2. Results

### 2.1. CROPGRO Tomato Model Calibration

The cultivar-specific parameters (CSPs) generated from the model calibration experiments for the variety Charger were presented in Table 1. These CSPs were compared with those previously calibrated for another commercial tomato variety (Florida 47) with similar characteristics by Boote et al. [12]. Although the estimated values for the CSPs for both varieties were generally similar, some slight variations were observed. The estimated value for the thermal time between plant emergence and flower appearance (EM.FL) was slightly higher for Charger (24.60) compared to Florida 47 (24.40). The thermal time between first flower and first fruit (FL.SH) was 2.50 for Charger and 2.20 for Florida 47. However, the thermal time between first flower and first seed (FL.SD) is shorter for Charger (16.00) compared to Florida 47 (19.00), while the maximum fraction of daily growth partitioned into fruit (XFERT) is slightly higher for Charger (0.80) compared to Florida 47 (0.78). Except for the mentioned parameters (EM.FL, FL.SH, FL.SD, and XFERT), the values for all other estimated parameters were similar for both varieties.

To access the accuracy of the calibrated CSPs for Charger, simulated values of days to anthesis and physiological maturity, biomass production, and tomato yield (Table 2) were compared to the corresponding field-observed values. A close agreement was observed between the simulated and the observed for all the four parameters evaluated. Both the anthesis and maturity dates were within ±1 d of the observed dates. The model slightly overpredicts both yield and biomass accumulation, although these values were withing acceptable range according to the evaluation statistics (Table 2).

### 2.2. CROPGRO Tomato Model Evaluation

**Figure 2:**
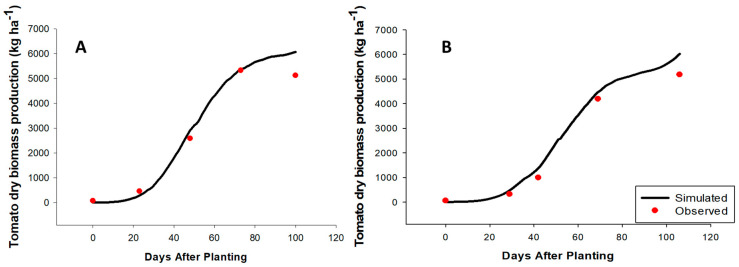
Results of the model calibration for the aboveground biomass accumulation during the fall 2016 (**A**) and spring 2017 (**B**) seasons of drip-irrigated fresh-market tomato production in Immokalee, Florida.

**Figure 3:**
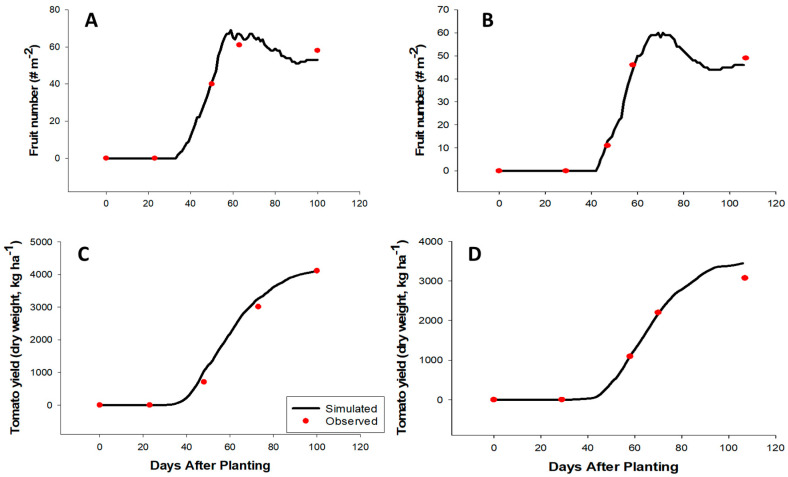
Results of the model calibration for fruit number during the fall 2016 (**A**) and spring 2017 (**B**) seasons and yield during the fall 2016 (**C**) and spring 2017 (**D**) seasons of drip-irrigated fresh-market tomato production in Immokalee, Florida.

After calibration, the model was able to predict anthesis and physiological maturity dates (Table 3), aboveground biomass accumulation, fruit number, and tomato yield (Table 4) for both the spring (Feb. to May) and fall (Aug. to Dec.) seasons. The individual variables—biomass (Figure 2), fruit number (Figure 3A,B), and yield (Figure 3C,D)—collected (under field conditions) at each of the tomato growth stages during both the fall and spring seasons were adequately tracked by the model; thus, they were indicative of model precision and accuracy. However, observed biomass accumulation was lower than the model prediction at the last growth stage (end of the season) during both seasons (fall (Figure 2A) and spring (Figure 2B)). These differences suggest that perhaps the magnitude of senescence under the field condition was greater than the model prediction. Hence, observed biomass (compared to the predicted value) was lower at the end of both growing seasons. Both the predicted and observed anthesis dates were 27 days after planting during the spring season while anthesis date was 22 (predicted) and 23 (observed) days after planting during a warmer fall season. The physiological maturity dates were 100 days after planting for both the predicted and simulated during the fall season and 106 and 105 days for the predicted and observed, respectively, during the spring season. Both the RMSE and *d*-Stat values obtained during the evaluation were similar to those reported by Boote et al. [12] on the effects of cardinal temperatures on multiple commercial tomato varieties grown in Florida.

### 2.3. Effects of High Temperatures on Tomato Yield and Growth

**Figure 4:**
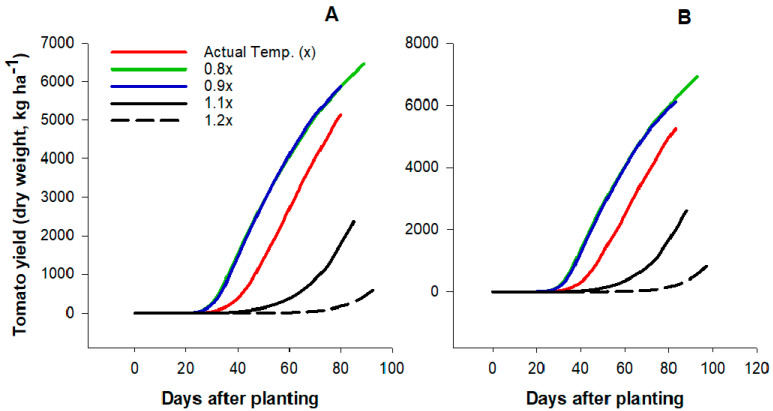
Effects of variations in air temperature on drip-irrigated fresh-market tomato yield of two commercial varieties Charger (**A**) and Florida 47 (**B**) in Immokalee, Florida.

Changes in temperature conditions affected tomato growth and productivity. At harvest, tomato fruit yield for both varieties decreased as air temperatures increased (Figure 4). Compared to the actual or current temperature condition (AT), tomato yield (average of the two varieties) was 16 and 29% higher at lower temperature regime (0.9x and 0.8x, respectively) and 52 and 85% lower at higher the temperatures (1.1x and 1.2x, respectively). Although a similar magnitude of temperature changes from the actual condition (i.e., 10% or 20% above or below) was evaluated in this study, the percentage changes (high or low) in yield were greater under the higher temperatures compared to the lower regimes.

**Figure 5:**
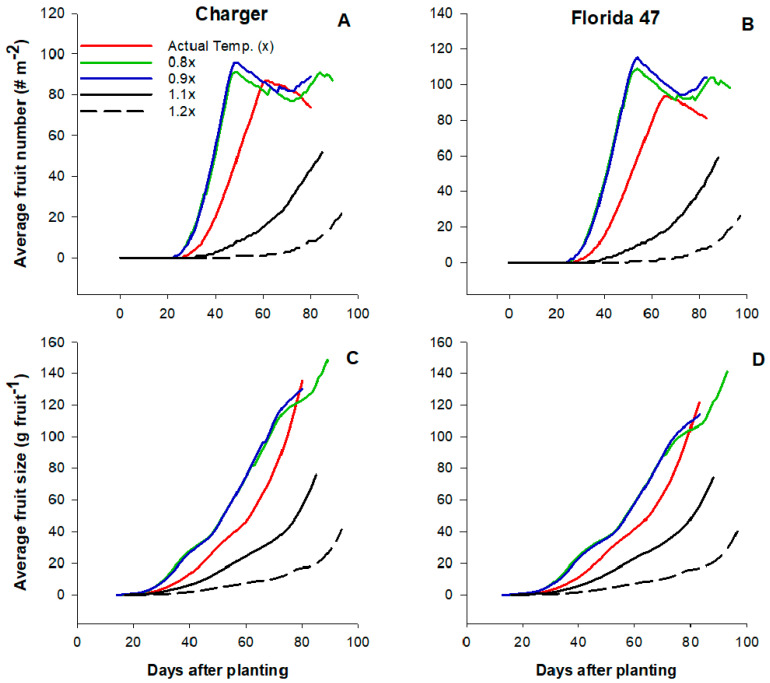
Effects of variations in air temperature on tomato fruit number (**A**,**B**) and fruit size (**C**,**D**) of two drip irrigated commercial fresh market tomato varieties (Charger and Florida 47) in Immokalee Florida.

**Figure 6:**
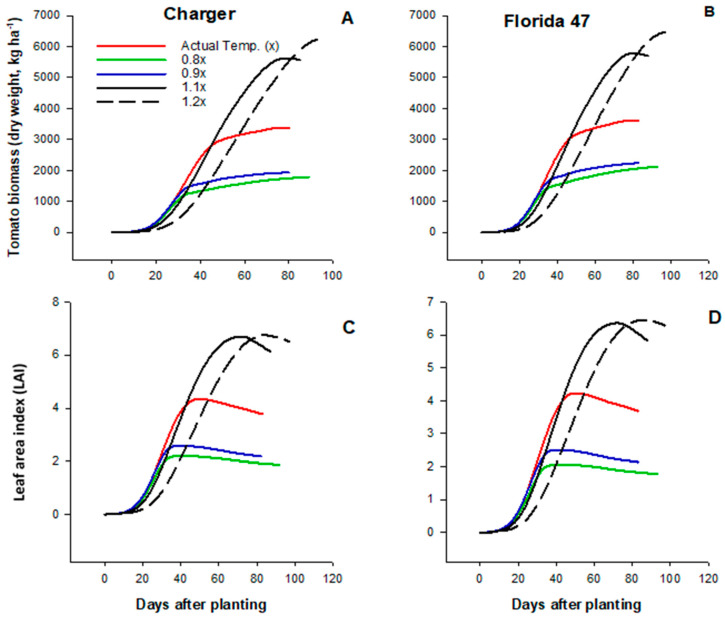
Effects of variations in air temperature on tomato biomass production (**A**,**B**) and leaf area index (**C**,**D**) of two drip irrigated commercial fresh market tomato varieties (Charger and Florida 47) in Immokalee Florida.

The simulated effects of temperature on tomato fruit number and fruit size were presented in Figure 5. The individual fruit size or weight under the current temperature regime was similar to the lower temperature conditions; however, average fruit number (77 fruit m^−2^) was lower under the actual temperature condition compared to the lower regimes at 97 and 93 fruits m^−2^ for 0.8x and 0.9x, respectively. Although the current temperature condition had no major effects on fruit size, both fruit size and number were significantly reduced as temperature increased further above the current condition (1.1x and 1.2x). Compared to the current temperature condition, average fruit number and fruit size decreased by 43% and 74%, respectively, under 1.1x and 1.2x conditions. Contrary to the yield parameters, tomato biomass production increased with temperature regardless of variety. Both tomato dry matter accumulation (Figure 6A,B) and leaf area index (LAI, Figure 6C,D) were highest at higher temperature conditions (1.1x and 1.2x). Under 0.8x and 0.9x conditions, average biomass accumulation and LAI were 46% and 54%, respectively, lower compared to the AT conditions. Higher dry matter production with increasing temperature was not a direct effect of increasing temperature but due to lower fruit production.

### 2.4. Effects of Planting Dates on Tomato Yield and Season Length

**Figure 7:**
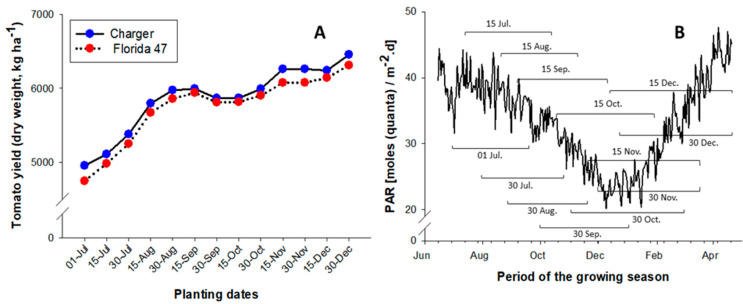
Effects of planting dates on drip irrigated fresh market tomato yield (**A**) and photosynthetic active radiation [PAR, (**B**)] collected from Florida Automated Weather Network during the growing seasons at different planting dates in Immokalee Florida.

To reduce the impact of heat stress during the growing season, several (13) planting dates were evaluated from July to December. The model was simulated for both Charger and Florida 47 tomato varieties using the actual weather condition from 2002 to 2017 for Immokalee Florida, results of which were presented in Figure 7A. These results indicated that tomato yield increased as planting date increased from July to December. As previously described in Figure 1, the average air temperatures at the study location was historically highest between July and August, thus yield was lowest for July 1 planting date (4851 kg ha^−1^) and highest at 30 December planting date (6383 kg ha^−1^). After this period, the air temperatures steadily declined towards December, hence, greater yield at the later planting dates with cooler temperatures. Although, the simulated tomato yields increased steadily as planting dates increased from July to December, a sudden drop in yield was observed for planting dates between late September to late October (30 September, 15 October, and 30 October). Photosynthetic active radiation (PAR) varied during each of the simulated growing seasons or planting dates (Figure 7B). PAR was lowest (about 20 mols m^−2^.d) between December and January and peaked from April to June (between 40 to 48 mols m^−2^.d). PAR decreased steadily from June to December and steadily increased from January to April.

Generally, season length increased as planting dates increased from July to December (Table 5). Although tomato yield was 32% higher for the late December planting date compared to early July, total season length increased by up to 32 days for the former (Table 5). Fall growing season may start as early as July for some growers, growing seasons for most farmers in south Florida typically starts between August and September. However, the study clearly demonstrated that there could be greater yield benefits for planting dates in November and December.

## 3. Discussions

The results of the model statistics for both calibration and evaluation as previously described indicated high *d*-Stat and low RMSE values thus, suggested a good agreement between the observed and the model simulated values. This indicated that the CSM-CROPGRO-Tomato model can be reliably used to evaluate tomato production under different cultural practices (especially irrigation and nutrient management) and climatic conditions. Thus, the model performed well in predicting phenological parameters (anthesis and physiological maturity dates), biomass accumulation and yield. Both the calibrated and evaluated model accuracy in this study agree with those previously reported for tomato under similar conditions in Florida [12]. Although the number of seasons used for both model calibration and evaluation for the Charger variety may be relatively small, a similar CSPs (hence, similar simulated results) with a previously calibrated Florida-47 variety by Boote et al. [12] from multiple seasons suggested a reliable calibration results for the Charger variety.

Temperature is an important abiotic stressor in tomato growth and development [12]. The impact of temperature-induced stress can become even more critical under high relative humidity [18], which is a typical condition during tomato growing seasons in south Florida. The results from this study suggest that tomato crop planted under the current temperature condition during Fall season in South Florida, may not attain their maximum genetic potential due to heat stress. Therefore, any additional warming of the climate may further reduce tomato productivity [15]. Lower yield under the current temperature condition was not as a result of individual fruit size, but the total number of fruits produced. This is because individual fruit size or weight under the current temperature regime was similar to the lower temperature conditions however, average fruit number (77 fruit m^−2^) was lower under the actual temperature condition compared to the lower regimes at 97 and 93 fruits m^−2^ for 0.8x and 0.9x respectively. Although the current temperature condition had no major effects on fruit size, both fruit size and number were significantly reduced as temperature increased above the current condition.

The model output for lower fruit number with higher temperature as observed in this study was assumed to have been caused by flower abortion or blossom drop as temperature increased. This is because plants are generally sensitive to temperature extremes during reproductive stages [19], resulting in fewer pollen production and reduced pollen viability and fruit set [20]. This is the reason why blossom drop during tomato growing season in Florida has been primarily associated with temperature extremes [8]. Blossom drop is related to lower yield because a higher rate of blossom drop would result in fewer fertilized flowers, hence, fewer fruit number at maturity. That is why Boote et al. [12] programmed the CSM-CROPGRO-Tomato model’s species files to reduce the rate of fruit-set and partitioning (to mimic reduced pollen viability) as temperature increases. Hence, dry matter partitioning to fruit reduces at air temperature rises above 28 °C and falls to zero at 34 °C [12]. Other possible factor that could have contributed to lower yield at higher temperature was increase in maintenance respiration resulting from elevated plant canopy temperature as ambient temperatures increases. Although with a minor effects on tomato yield, at higher temperatures, tomato plants tends to allocate a greater proportion of resources into physiological maintenance processes at the expense of reproduction as the canopy temperature increases, hence this condition may also contribute to a lower yield [12].

Although increase in temperatures could result in heat stress for tomato crop, the simulated increase in plant growth with high temperatures could as well be buffered by adequate plant water availability to meet crop evaporative demand. Since the model was simulated under a non-limiting water and nutrient conditions, the effects of heat stress reported in this study could be relatively lower compared to a similar temperature condition with limited available soil water and/or nutrient content. Similarly, the increasing biomass accumulation under high temperature could not be attributed to a direct effect of the elevated temperature on tomato growth. This is because under a high-temperature induced stress, tomato fruit set is significantly reduced, resulting in a higher partitioning of resources (carbon, water, and nutrient) into vegetative biomass compared to plants under optimum temperature condition with no heat stress [7,8,9,12,20].

The effects of planting dates indicated that both tomato yield and season length increased as planting date increased from July to December. Since the air temperature decreased with planting dates from July to December, therefore, the increase in tomato yield and season length were attributed to cooler temperatures during the later planting dates. The effects of temperatures on season length reported in this study were similar to those previously reported under similar growing conditions in south Florida [20]. The sudden drop in yield between late September and late October planting dates (September 30, October 15, and October 30) was attributed to lower density of PAR. Historically, PAR at the study location steadily declines from June to January and increased afterwards, therefore lower yield during this planting dates was either because a major proportion or entire growing season occurred during period of lower PAR compared to the other planting dates. Thus, planting date between 30th September and 30th October may not be ideal for tomato production in south Florida.

Therefore, due to lower yield during July and August planting dates and the potential effects of lower PAR during September and October in south Florida, planting dates between November and December could be considered the optimum for tomato production in south Florida. This is because the air temperatures are typically cooler during this period hence, lower impact of heat stress on general growth and productivity of open-field fresh-market tomato. Although the economics of the production system was not the focus of this study, it is important to recognize that due to a longer season, the production cost may slightly increase for the planting dates in November and December compared to July and August. However, this increase in cost may be insignificant as additional revenue due to higher yield may offset cost increase. Also, planting dates in November and December may increase the probability of frost damage during the production season and growers may not be able to achieve more than one production cycle from a given field within a production year.

## 4. Materials and Methods

### 4.1. Study Location

The study was conducted at the University of Florida Institute of Food and Agricultural Sciences (UF/IFAS), Southwest Florida Research and Education Center (SWFREC), in Immokalee, Florida (Latitude 26°27′44′′ N and longitude 81°26′36′′ W, elevation of 10.4 m above sea level). The air temperatures typically range from 11 to 35 °C and average annual precipitation of 965 to 1727 mm. The soil at the study location was classified as Spodosol (soil Order) and Immokalee fine sand (soil series) [21], with low runoff class, poor natural drainage and a relatively high saturated hydraulic conductivity (Ksat, 15.82 cm h^−1^) at the upper 15 cm of soil depth [22]. Located in the north of Collier County, Immokalee is famous for vegetable production, packaging, and distribution in Florida hence, an important community for tomato production in south Florida.

### 4.2. DSSAT CROPGRO-Tomato Model

The cropping system model CSM-CROPGRO-Tomato distributed by DSSAT (Decision Support System for Agricultural Transfer) version 4.70 [23,24] was used in this study. The DSSAT crop models integrate a number of different input systems such as soil, climate, and crop management systems with several other application programs [25]. DSSAT consists of 28 individually developed crop-specific models [26] under one platform for cereals, legumes, vegetables, fiber, root, oil, forage, fruit, and sugar/energy crops. Hence, the CSM has been extensively used in numerous studies to evaluate management practices and the impacts of environmental variables on row and vegetable crops from a wide range of geographical and environmental conditions [12,26,27,28,29,30,31]. The CSM-CROPGRO-Tomato model was calibrated under Florida conditions for tomato growth and production [12], and it was used in this study to determine the impacts of the current air temperature conditions on tomato production in south Florida. The current CSM-CROPGRO-Tomato model used in this study was recently modified by Boote et al. [12] to update the cardinal temperature parameters that influence both the vegetative and reproductive growth of tomato crop. This model requires soil, crop, and weather conditions during the growing season as input parameters. The soil parameters include legacy soil nutrient (N-P-K) concentration, soil water properties (drained upper limit (DUL) and drained lower limit (DLL)), hydraulic conductivity, saturated water content, soil temperature, soil organic carbon concentration, soil texture, color, and bulk density. The model uses daily weather data such as maximum and minimum air temperatures, solar radiation, relative humidity, precipitation, and wind speed. The crop parameters include the anthesis and maturity dates, dry matter accumulation, and yield for the estimation of cultivar-specific coefficients needed for the model outputs [12]. In addition to soil, crop, and weather information, crop management data are required as an additional model input. Crop management data include planting details, irrigation application schedules, nutrient amount and application time, anthesis and maturity dates, biomass sampling data, and dates, as well as crop harvest data.

### 4.3. Climate, Soil, and Crop Data Collection

The CSM-CROPGRO-Tomato model was parameterized using field data to simulate the impact of temperature on tomato growth and yield. Data input followed the minimum input data required for DSSAT to simulate crop growth [24]. This study was conducted with 14 years of (2002–2015) observed daily weather information for the study location. The weather information, including photosynthetic active radiation (PAR), was obtained from the FAWN (Florida Automated Weather Network) station located within 0.5 km of the study location. Soil data (Table 1) input such as organic carbon, pH, cation exchange capacity, and soil water properties (saturation, lower drain limit, and upper drain limit) were obtained from a previous study by Kadyampakeni et al. [22] on the same soil series adjacent to the study location. Other soil properties such as series name, color, drainage, runoff, and profile description were obtained from the USDA/NRCS soil survey data [21].

Crop parameters such as tomato yield and growth or biomass accumulation data were collected from a two-season (fall 2016 and spring 2017) field study. The planting and fruit maturity dates were 8 September and 28 November 2016; and 7 February and 21 May 2017, for the fall and spring seasons, respectively. During each season, tomato seedlings (variety Charger by Sakata. Morgan Hill, CA, USA) were transplanted at about five weeks after germination at 60 cm plant spacing in a single row about 21 days after bed preparation. For each season, one sampling plot consisted of 90 plants at an area of about 67 m^2^ equivalent to a density of 9410 plant per ha. In both seasons, the tomato water requirement was determined using SmartIrrigation (SI) Vegetable as described by Ayankojo et al. [32]. SI Vegetable is an ET-based irrigation scheduling method developed as a smart phone application. Irrigation scheduling from SI uses ETo from the FAO Penman–Motheith procedure [33] and *K_c_* to determine crop water requirement [34]. SI is an irrigation decision support system that provides users with irrigation schedules based on real-time, location-specific weather data [34]. The SI technology has been extensively used for irrigation scheduling in crops such as citrus (*Citrus sinencis* L.), cotton (*Gossypium* spp. L.), turf grass, strawberry (*Fragaria* spp.), avocado (*Persea americana*) [34], tomato (*Solanum lycopersicum* L.) [32,35,36] and watermelon (*Citrilus lanatus* (Thumb.) Matsun. and Nakai) [37]. The SI scheduling method has not only been reported to significantly increase water savings but also increase crop yield [32,37] and improve iWUE [35,38] as well as increase in crop nutrient recovery by maintaining nutrient within the root zone [35]. More detailed information on SI technology is available at https://smartirrigationapps.org/.

Nutrient application during both seasons followed the grower’s standard for open-field fresh-market tomato production in Florida [39] with total N, P_2_O_5_, and K_2_O at 269, 14, and 224 kg ha^−1^, respectively. Tomato biomass data were collected at each of the five phenology stages of open-field tomato crop (Table 6). During both seasons, both biomass and yield data were collected (*N* = 4 at each sampling period) from four replicate plots. Tomato biomass at each sampling stage was separated into its component parts (leaf, stem, root, and fruit). After each sampling period, samples were oven-dried and weighed to determine dry matter accumulation. Yield sampling was conducted by removing green mature and color break fruits from 10 treatment-representative plants per plot. Fruit harvest was conducted three times per season at approximately two-week intervals during each production season. Tomato yield were presented based on dry matter basis from the oven-dried fruit samples collected at harvest.

### 4.4. Model Calibration and Evaluation

Model evaluation was conducted using two commercial tomato varieties (Charger and Florida 47) commonly used among growers in Florida. The variety Florida 47 has been previously calibrated by Boote et al. [12] in DSSAT for the conditions in Florida, hence no further calibration was required. However, variety Charger was calibrated using the data from the previously described two-season study conducted during the fall 2016 and spring 2017 seasons on drip-irrigated fresh-market tomato with plastic mulch. Both the growth and phenological development controlling parameters were estimated using the generalized likelihood uncertainty estimation (GLUE) (available within the DSSAT model) with 6000 number of runs. Additionally, the generated coefficients were manually checked for adjustment using the sensitivity analysis tool in DSSAT version 4.7. GLUE uses a Bayesian parameter estimation procedure to estimate CSPs [40]. GLUE will “discretize the parameter space by generating a large number of parameter values from the prior distribution” [40]. It then used field observations to estimate the likelihood values for each parameter after which an empirical posterior distribution is generated using the Bayes equation [40]. GLUE used the data collected (anthesis, maturity, harvest dates, and leaf, stem, root, and fruit dry weights) during both growing seasons to estimate the coefficients for the tomato cultivar used. In this study, the estimated parameters included the eighteen CSPs required by the CROPGRO-Tomato model in DSSAT 4.7. To ensure optimum model operations, the model was calibrated using data from a well-watered (100% ET_c_) and well-fertilized (previously described) field study collected during the two growing seasons.

After calibration, the generated CSPs were then evaluated using independent data from both the 2016 and 2017 growing seasons with different total N application rate (180 kg ha^−1^) different from those used during model calibration. During model evaluation, the index of agreement (*d*-Stat) [41], and root-mean-square error (RMSE) [42] were used as the statistical indices to determine model accuracy. The combination of coefficients that resulted in the highest *d*-Stat and lowest RMSE between the observed data in the field and the simulated values were selected as the final coefficients.

(1) $$d=1-\left[\frac{\sum_{i\;=\;1}^{N}{\left(\hat{Y}i-\bar{Y}i\right)}^{2}}{\sum_{i\;=\;1}^{N}{\left(|\hat{Y}i\;-\bar{Y}\left|\;+\;\right|Yi-\bar{Y}i\;\right)}^{2}\;}\right],\;0\leq d\geq 1$$

(2) $$RMSE\;=\;\sqrt{\frac{\sum_{i\;=\;1}^{N}{\left(\hat{Y}i-\bar{Y}i\right)}^{2}}{N}}$$

where *Yi* is the observed value, $\hat{Y}$ is the simulated value, $\bar{Y}i$ is the average of the simulated values, $\bar{Y}i$ is the average of the observed values, and *N* is the number of observations.

### 4.5. Description of the Simulated Treatments

The successfully calibrated and evaluated CSPs were later used for the simulation of the effects of air temperature on tomato growth and production in south Florida under the assumption that water and nutrients were not limited. In this study, five temperature regimes were evaluated (Table 7). The first treatment (AT) was simulated using the 14 years (2002–2015) of actual weather conditions collected at the study location. All other treatments were conducted based on a percentage (−20%, −10%, +10%, +20%) of the actual weather condition to simulate lower and higher temperature regimes, respectively. The AT treatment was used as the baseline to which the other treatments were compared. The lower temperature regimes (−20% and −10%) were simulated to compare the potential severity of heat stress on tomato growth and productivity under the AT condition. The higher temperature regimes (+10%, +20%) were simulated to determine the potential effects of further increase (away from the AT condition) in temperature condition for the future. The simulated temperature conditions were within the projected range for the potential increase in global mean air temperature (2.6 and 4.8 °C) by the end of the twenty-first century [2].

To determine the optimum planting date for tomato during the fall season in south Florida, additional simulation was conducted by evaluating the effects of planting dates on tomato productivity. Both tomato varieties previously described were used in this simulation. Since planting season can start as early as July to early August, this study evaluated 13 planting dates (at 15 days intervals) from July 1 to December 30. The optimum range of planting dates were determined based on maximum tomato yield or productivity. Since the current version of CSM-CROPGRO-Tomato 4.7 does not consider the effect of plastic mulch on soil nutrient and water conditions, the model was modified to ignore precipitation during the calibration, evaluation, and simulation procedures. Therefore, soil water and nutrient conditions were only affected by irrigation and not precipitation. This was done to: (1) obtain soil water content similar to the actual field conditions, (2) simulate the effects of plastic mulch on soil water and nutrient conditions, and (3) prevent incorrect nitrogen (N) leaching model output associated with rainfall.

## 5. Conclusions

Tomato production under the current weather condition during the fall growing season in south Florida may be exposed to high temperature induced stress. This condition could result in a significant yield reduction compared to lower temperature conditions. The primary effects of higher temperature in the tomato crop reduced fruit number and yield due to lower pollen viability and fruit set. With the predicted increase in global temperature for the future, a further increase in temperature suggests a much lower tomato productivity in south Florida. Therefore, modifying planting dates from the current July and August to November and December could be an important cultural practice to reduce heat stress and significantly increase tomato productivity in south Florida.

## Figures and Tables

**Table 1:** Genetic coefficients of tomato (var. Charger) calibrated in DSSAT (Decision Support System for Agricultural Transfer) against a previously calibrated FL 47 tomato cultivar.

Code	Description	Charger	FL.47
EM.FL	Time between plant emergence and flower appearance (R1) (photothermal days)	24.60	24.4
FL.SH	Time between first flower and first pod (R3) (photothermal days)	2.50	2.20
FL.SD	Time between first flower and first seed (R5) (photothermal days)	16.00	19.00
SD.PM	Time between first seed (R5) and physiological maturity (R7) (photothermal days)	45.01	45.20
FL.LF	Time between first flower (R1) and end of leaf expansion (photothermal days)	52.00	52.00
LFMAX	Maximum leaf photosynthesis rate at 30 °C, 350 vpm CO_2_, and high light (mg CO_2_/m^2^-s)	1.36	1.36
SLAVR	Specific leaf area of cultivar under standard growth conditions (cm^2^/g)	300.0	300.0
SIZLF	Maximum size of full leaf (three leaflets) (cm^2^)	300.0	300.0
XFRT	Maximum fraction of daily growth that is partitioned to seed + shell	0.80	0.78
WTPSD	Maximum weight per seed (g)	0.004	0.004
SFDUR	Seed filling duration for pod cohort at standard growth conditions (photothermal days)	26.00	26.00
SDPDV	Average seed per pod under standard growing conditions (#/pod)	300.0	300.0
PODUR	Time required for cultivar to reach final pod load under optimal conditions (photothermal days)	55.00	55.00
THRSH	Threshing percentage. The maximum ratio of (seed/(seed+shell)) at maturity. Causes seed to stop growing as their dry weight increases until the shells are filled in a cohort.	8.50	8.50
SDPRO	Fraction protein in seeds (g(protein)/g(seed))	0.30	0.30
SDLIP	Fraction oil in seeds (g(oil)/g(seed))	0.05	0.05

**Table 2:** Simulated and observed mean values for anthesis, maturity, fruit yield, and biomass production at physiological maturity and their respective statistical indices of tomato variety Charger.

Parameter	Simulated	Observed	RMSE	*d*-Stat
Days to anthesis	24	25	0.71	0.98
Days to physiological maturity	103	102	0.71	0.99
Dry biomass production (kg ha^−1^)	6635	6419	217	0.97
Fruit yield (kg ha^−1^)	4310	4231	124	0.99

**Table 3:** Result of the model calibration for anthesis and maturity dates during the fall 2016 and spring 2017 seasons of drip irrigated fresh market tomato production in Immokalee, Florida.

Treatment	Simulated	Measured	RMSE	*d*-Stat
Anthesis date				
Fall 2016	22	23	0.71	0.98
Spring 2017	27	27		
Physiological maturity date				
Fall 2016	100	100	1.17	0.97
Spring 2017	106	105		

**Table 4:** Root-mean-square error (RMSE) and Willmott *d* index (*d*-Stat) for biomass production, fruit weight, and fruit number after calibration.

Season	Aboveground Biomass		Total Fruit Weight		Fruit Number	
	RMSE	*d*-Stat	RMSE	*d*-Stat	RMSE	*d*-Stat
Fall 2016	456	0.99	248	0.99	15.12	0.78
Spring 2017	440	0.96	112	0.98	2.00	0.99

**Table 5:** Effects of planting dates on changes in yield and length of growing season of two commercial tomato varieties in Immokalee Florida.

Planting Dates	Change in Yield (%) ^Z^	Change in Season Length (days) ^y^	Change in Yield (%)	Change in Season Length (days)
	Charger		Florida 47	
01-July	−23.3	0	−24.8	0
15-July	−20.9	0	−21.1	0
30-July	−16.7	0	−16.8	0
15-August	−10.2	+2	−10.1	+2
30-August	−7.5	+5	−7.2	+5
15-September	−7.2	+10	−5.9	+10
30-September	−9.1	+16	−7.9	+16
15-October	−9.1	+25	−7.8	+25
30-October	−7.2	+32	−6.5	+31
15-November	−3.0	+34	−3.7	+33
30-November	−3.0	+34	−3.7	+33
15-December	−3.3	+32	−2.7	+32
30-December	0	+29	0	+28

^Z^ changes in tomato fruit production with respect to 30 December the planting date with the highest yield. ^y^ changes in length of growing season with respect to July, the shortest growing season.

**Table 6:** Description of the tomato developmental stages used as the guide for sampling collection.

Stage	Description
1	From transplant until nine or more leaves on the main shoot unfolded
2	From first flower open until the eighth inflorescence first flower opened
3	From the ninth inflorescence first flower opening until its fruits reach typical size but no color changes
4	Fruit maturity until first harvest
5	After first harvest until last harvest

**Table 7:** Simulated maximum (daytime) temperatures.

Treatment #	Treatment Description ^1^	Avg. Daily Max. Temp.	Avg. Daily Min. Temp.
		°C	
T1	Actual Temperature (AT)	32	23
T2	AT*0.8 (0.8x)	26	18
T3	AT*0.9 (0.9x)	29	20
T4	AT*1.1 (1.1x)	34	25
T5	AT*1.2 (1.2x)	38	28

^1^ AT was simulated using 14 years (2002–2015) of actual weather conditions collected at the study location. All other treatments were conducted based on a percentage of the AT condition to simulate lower and higher temperature regimes.
